# Evaluation of Pregnant Women’s Perspectives on Root Canal Treatment: A Cross-Sectional Study

**DOI:** 10.3390/healthcare14091138

**Published:** 2026-04-24

**Authors:** Ayfer Atav, Aysenaz Gunes, Emre Ovsay, Celalettin Topbaş

**Affiliations:** 1 Department of Endodontics, Faculty of Dentistry, Istanbul Atlas University, Istanbul 34403, Türkiye; emreovsay@gmail.com; 2 Department of Clinical Sciences, Faculty of Dentistry, Istinye University, Istanbul 34396, Türkiye; aysenaz.gunes@istinye.edu.tr; 3 Department of Stem Cell and Tissue Engineering, Institute of Graduate Education, Istinye University, Istanbul 34396, Türkiye; 4 Department of Endodontics, Faculty of Dentistry, University of Health Sciences, Istanbul 34668, Türkiye; dt.c.topbas@gmail.com

**Keywords:** cross-sectional study, dental treatment, endodontics, pregnant women

## Abstract

**Highlights:**

**What are the main findings?**

- The vast majority of participants believed that treating toothache and dental infections during pregnancy is important for both maternal and fetal well-being; however, a considerable proportion still perceived root canal treatment as unsafe at any stage of pregnancy.
- Nearly half of pregnant women who experienced toothache during pregnancy neither used analgesics nor sought dental care. Additionally, most pregnant women who believed they required root canal treatment preferred to postpone the procedure until after delivery, revealing a gap between knowledge and treatment acceptance.

**What are the implications of the main findings?**

- The findings suggest that concerns and misconceptions regarding the safety of dental procedures during pregnancy may contribute to delayed or avoided dental care.
- Clear communication and evidence-based guidance from both dentists and antenatal care providers may help improve pregnant women’s awareness of the safety and necessity of dental treatment during pregnancy.
- Strengthening interdisciplinary collaboration and patient education may facilitate timely dental care and reduce unnecessary postponement of essential endodontic treatment.

**Abstract:**

**Objectives**: Oral healthcare and regular dental follow-up are important during pregnancy, as maternal oral health may have important implications for both maternal and fetal well-being. However, dental attendance remains low. Therefore, this cross-sectional e-survey aimed to explore pregnant women’s dental pain management strategies, their perceptions of endodontic treatment, and avoidance of specific endodontic procedures during pregnancy. **Methods**: An 18-item online questionnaire was administered to 130 pregnant women. Data were collected on dental attendance, experiences of toothache, use of analgesics or antibiotics, and perceptions of the safety of dental anesthesia and radiographic procedures during pregnancy. Statistical analyses were conducted using Chi-square or Fisher’s exact tests and multivariate logistic regression (*p* < 0.05). **Results**: The mean age of participants was 32 years. Only 26.15% reported attending regular dental visits during pregnancy. Among participants who experienced toothache, 51.52% used analgesics and 1.54% used antibiotics. Although 92.31% believed dental infections should be treated during pregnancy, concerns regarding dental procedures were common; 76.92% considered dental radiography unsafe and only 50% considered local anesthesia safe. Multivariate analysis showed that the number of pregnancies was associated with dental visits during pregnancy (*p* = 0.048), age with analgesic use (*p* = 0.018), and education level with perception of dental radiography safety (*p* = 0.013). **Conclusions**: Despite awareness of the importance of treating dental infections, pregnant women may delay endodontic care during pregnancy, highlighting a need for improved patient education and clearer clinical guidance.

## 1. Introduction

Pregnancy is a period marked by physiological and hormonal changes that can influence oral health [1,2]. The changes in the female body during pregnancy may cause oral manifestations such as gingivitis, pyogenic granuloma, tooth decay, perimyolysis, xerostomia, and tooth mobility [3,4,5,6]. These oral conditions may negatively impact the quality of life of pregnant women. During pregnancy, dental conditions such as caries, pulpitis, and periapical infections may cause pain, discomfort, and functional limitations [2,7,8]. Moreover, mothers’ oral health can influence pregnancy outcomes. For instance, periodontal diseases and a heavy oral microbial burden during pregnancy have been associated with obstetrical complications like low birth weight, preterm birth, and preeclampsia [7]. Considering the oral conditions and their possible results, appropriate oral healthcare and regular dental follow-up during pregnancy are essential [9].

As maternal oral health status is closely associated with pregnancy outcomes and fetal well-being, pregnant women’s awareness and perceptions regarding oral and dental health gain critical importance [1,9]. If not managed appropriately, dental infections may progress and eventually require urgent intervention. For this reason, maintaining oral health and addressing dental problems in a timely manner are important aspects of care during pregnancy [9,10].

Endodontic treatment is often required to manage dental pain and infection originating from pulpal and periapical tissues [10,11]. Available evidence indicates that root canal treatment can be performed safely during pregnancy when standard clinical precautions are followed [11,12]. The use of local anesthesia, routine infection control measures, and dental radiographic procedures with appropriate shielding are generally regarded as safe for both the mother and the fetus. Nevertheless, endodontic procedures are frequently approached with caution during pregnancy, largely due to concerns related to anesthetic use and radiographic exposure [10,11].

Despite professional recommendations supporting the provision of necessary dental care during pregnancy, avoidance or postponement of treatment remains common. Concerns about potential harm to the fetus, limited awareness of the safety of dental procedures, and variable advice from healthcare providers may influence pregnant women’s decisions [2]. Previous studies have highlighted gaps in knowledge and ongoing misconceptions regarding dental treatment during pregnancy [2,13,14]. However, evidence specifically addressing pregnant women’s perceptions of endodontic treatment and their dental pain management strategies remains limited [2].

In Türkiye, evidence regarding pregnant women’s attitudes toward endodontic treatment and their dental pain management behaviors during pregnancy remains limited. Most previous studies have focused on knowledge and awareness related to oral health and dental care in this population [15,16,17,18]. In contrast, limited evidence is available regarding pregnant women’s experiences of dental problems and their attitudes toward treatment decisions during pregnancy. Therefore, this cross-sectional study aimed to evaluate pregnant women’s strategies for managing dental pain, their perceptions of endodontic treatment, and their tendency to avoid endodontic procedures during pregnancy.

## 2. Materials and Methods

### Survey Design and Data Collection

The survey questions were developed based on similar studies conducted in other countries [19,20,21]. Prior to the main survey, the questionnaire was pilot-tested in 15 pregnant women to evaluate the clarity and comprehensibility of the survey items. Based on feedback obtained during the pilot phase, minor wording adjustments were made to improve clarity. The pilot participants were not included in the final analysis. In addition, the questionnaire was reviewed by researchers experienced in endodontics and oral health research to ensure face and content validity. The internal consistency of the questionnaire was evaluated using Cronbach’s alpha coefficient, which was 0.88, indicating good reliability.

This study was conducted as a cross-sectional, questionnaire-based investigation using an online survey developed with Google Forms. Participation was voluntary and anonymous, and informed consent was obtained from all participants. To prevent multiple submissions, participants were required to sign in with a Google account; however, email addresses were not collected, and no personally identifiable information was recorded. All responses were stored in a secure database accessible only to the research team. The confidentiality and anonymity of all responses were strictly maintained throughout the data collection and analysis process.

The survey link was distributed electronically via WhatsApp to pregnancy-related community support groups, enabling voluntary participation of women with confirmed pregnancies. These groups consist of pregnant women from different regions of Türkiye who participate in online pregnancy support communities. Inclusion criteria included women with a confirmed pregnancy and the ability to complete the online questionnaire. Participants were invited to complete an 18-item questionnaire (Table 1). An a priori power analysis was performed using G*Power software version 3.1.9.7 (Heinrich-Heine-Universität Düsseldorf, Germany) for chi-square tests. Assuming a medium effect size (Cohen’s w = 0.30), α = 0.05, power = 80%, and df = 3 for the primary contingency table analyses, the minimum required sample size was calculated as 122 participants. The assumed effect size was based on a previous study conducted by Pattanshetti et al. [21]. Considering the possibility of non-response, the survey invitation was distributed to 500 pregnant women, and 130 completed questionnaires were included in the final analysis.

Before participation, all pregnant women were informed about the aim of the survey, namely, to evaluate their perspectives on endodontic treatment, and were informed that the collected data would be analyzed statistically. As part of the study design, participants were not provided with any educational information regarding dental treatments during pregnancy.

The questionnaire covered dental attendance habits, experiences of toothache during pregnancy, whether dental care was sought due to toothache, and the use of analgesics or antibiotics during pregnancy. In addition, participants were asked about their perceptions of the safety of dental radiography when appropriate protective measures are used, as well as the safety of dental anesthesia during pregnancy. The questionnaire also included socioeconomic indicators such as educational level and employment status to better characterize the study population (Table 1).

Data analysis was performed using SciPy software (version 1.2.3). Descriptive statistics were presented as absolute numbers (n) and percentages (%). Depending on the nature of the variables, statistical comparisons were carried out using the Chi-square test or Fisher’s exact test where appropriate. Multivariable logistic regression analyses were performed to identify factors associated with dental care utilization and perceptions during pregnancy. The dependent variables were defined as follows: (1) presence of dental visit during pregnancy, (2) perceiving root canal treatment as safe during pregnancy, (3) analgesic use due to dental pain, (4) perceiving dental radiography as safe during pregnancy, and (5) perceiving dental anesthesia as safe during pregnancy.

For each model, the same set of independent variables was included: age, employment status (employed vs. not), education level, number of pregnancies, and gestational month. All candidate predictors were pre-specified based on clinical relevance and were entered simultaneously into the multivariable models without prior univariate screening.

Odds ratios (ORs) with 95% confidence intervals (CIs) and corresponding *p*-values were calculated for all variables. To improve transparency and interpretability, all candidate predictors, including non-significant variables, were reported in the results.

Although multiple outcomes were analyzed, each model addressed a distinct clinical question. Therefore, formal adjustment for multiple comparisons (e.g., Bonferroni correction) was not applied. However, all *p*-values are reported to allow transparent interpretation of the results, and findings were interpreted cautiously. A *p*-value of less than 0.05 was considered statistically significant.

## 3. Results

The mean age of the participants was 32 years. Most of the pregnant women had completed university or higher education and were employed as working professionals (Table 2). For the majority of participants (77.69%), the current pregnancy was their first (Table 3). At the time of the survey, most women (95.38%) were in the second or third trimester of pregnancy (Table 4).

Regarding regular dental check-ups before pregnancy, 46.92% of participants reported attending, whereas 53.08% indicated that routine dental visits were not a priority even before becoming pregnant. During pregnancy, dental attendance decreased further, with only 26.15% of participants reporting a visit to a dentist (Table 5). Among participants who sought dental care during pregnancy, periodontal problems (30.23%) were the most commonly reported reasons for attendance (Table 6).

Most participants (92.31%) believed that the treatment of toothache and dental infections during pregnancy is necessary, yet 36.15% identified root canal treatment as not safe at any stage of pregnancy. Root canal treatment during pregnancy was reported by 3.85% of the participants (Table 7).

In multivariable logistic regression analyses, the number of pregnancies was the only variable significantly associated with the presence of dental visits during pregnancy, with higher parity associated with increased likelihood of dental visits (OR: 1.19, 95% CI: 1.01–2.58, *p* = 0.048). Age, employment status, university education, and gestational month were not significantly associated with dental visit (Table 8).

For the perception of root canal treatment as safe during pregnancy, none of the evaluated variables, including age, employment status, education level, number of pregnancies, and gestational month, showed a statistically significant association (Table 8).

Analgesic use due to dental pain was significantly associated with age, with older participants being more likely to use analgesics (OR: 1.20, 95% CI: 1.03–1.39, *p* = 0.018). No significant associations were observed for employment status, education level, number of pregnancies, or gestational month (Table 8).

Regarding the perception of dental radiography as safe during pregnancy, both age (OR: 1.14, 95% CI: 1.01–1.29, *p* = 0.047) and education level were significantly associated with the outcome. Specifically, non-university graduates were less likely to perceive dental radiography as safe (OR: 0.57, 95% CI: 0.37–0.89, *p* = 0.013). Other variables were not significant (Table 8).

Similarly, for the perception of dental anesthesia as safe during pregnancy, education level remained a significant factor, with non-university graduates showing lower likelihood of perceiving anesthesia as safe (OR: 0.63, 95% CI: 0.43–0.94, *p* = 0.022). Age, employment status, number of pregnancies, and gestational month were not significantly associated with this outcome (Table 8).

The results of the analyses conducted among the 33 participants who answered “yes” to the question “Do you need root canal treatment?” are presented in Table 9. Participants who answered “yes” to the question “Did you experience severe toothache during pregnancy?” were significantly associated with avoidance or postponement of root canal treatment. Participants who did not report severe toothache were significantly more likely to avoid or postpone root canal treatment (90.9%, *p* = 0.021) (Table 9). Among participants who reported using analgesics for toothache, 58.8% did not avoid or postpone root canal treatment. In contrast, 87.5% of those who did not use analgesics reported postponing treatment (*p* = 0.006). (Table 9). Among pregnant women requiring root canal treatment, 48.48% reported not using analgesics during pregnancy and 72.73% of them marked to postpone treatment after delivery (Table 10).

Participants were asked about their perceptions of procedures involved in endodontic treatment, including the administration of local anesthesia and the use of dental radiography. While half of the participants considered the use of local dental anesthesia to be safe during pregnancy, only 23.08% regarded dental radiographic procedures performed with appropriate protective measures as safe (Table 11).

## 4. Discussion

Pregnancy is associated with physiological and hormonal changes that increase susceptibility to oral conditions such as gingival inflammation, dental caries, and pulpal or periapical pathology [1,2]. These oral conditions may cause pain, discomfort, and functional limitations in pregnant women [2,7,8]. Of utmost importance, maternal and fetal well-being may be negatively influenced by poor oral health conditions [7]. Consistent with previous literature, the findings of the present study may indicate that oral health challenges during pregnancy remain clinically relevant, yet participants reported limited utilization of dental and endodontic services despite established recommendations supporting timely dental care during pregnancy [22,23,24].

The reluctance to seek dental care is especially concerning given the potential consequences of untreated oral disease for both maternal and fetal health. Dental infections and inflammatory conditions have been associated with adverse pregnancy outcomes [1,25]. However, participants in the present study reported concerns regarding the safety of dental procedures during pregnancy, which may contribute to delays in seeking dental care. In the present study, concerns related to local anesthesia, radiographic imaging, and medication use emerged as prominent barriers to care, which suggests that concerns or uncertainty regarding procedural safety persist among participants.

Perceptions regarding the safety of local anesthesia and dental radiography were particularly influential. Only half of the participants considered local anesthesia to be safe during pregnancy, despite evidence supporting the safe use of commonly administered anesthetics such as lidocaine and prilocaine when appropriate precautions are taken. Current clinical guidelines, including those of the American College of Obstetricians and Gynecologists (ACOG), indicate that necessary dental procedures requiring local anesthesia can be safely performed during pregnancy when administered at recommended doses [26]. Commonly, 2% Lidocaine with epinephrine (1:100,000 or 1:200,000) is considered the anesthetic of choice in dental practice due to its favorable safety profile during pregnancy. The maximum recommended dose of lidocaine is 4.5 mg/kg without epinephrine (maximum 300 mg) and 7 mg/kg with epinephrine (maximum 500 mg). When used within these recommended limits, dental local anesthetics are not considered to pose a significant risk to the fetus [27]. Adequate anesthesia is essential for effective endodontic treatment, and uncertainty in this regard may discourage women from pursuing definitive care [7]. Similarly, although diagnostic radiography is a critical component of endodontic decision-making and is considered safe with modern techniques and protective measures, hesitation toward radiographic procedures persisted among participants in this study [7]. These findings highlight the importance of improving patient education regarding the safety of dental procedures during pregnancy and strengthening communication between dentists and obstetric healthcare providers.

Pain management strategies further reflect treatment avoidance behavior. A considerable proportion of participants who experienced severe toothache did not use analgesics or seek dental care and endured the dental pain. Half of pregnant women who experienced severe toothache visited a dentist to determine the cause of their pain. However, nearly three out of four of women who required root canal treatment postponed the procedure until after delivery. This trend indicates that seeking dental consultation does not necessarily translate into acceptance of the treatment. This observation may suggest a discrepancy between participants’ reported awareness of oral health importance and their treatment-seeking attitude. Because the subgroup of participants requiring root canal treatment was relatively small (n = 33), these findings should be interpreted cautiously and considered exploratory.

A notable observation in this study is the discrepancy between participants’ reported awareness of the importance of oral health and their self-reported treatment-seeking behavior. Although most participants considered dental treatment during pregnancy to be necessary, a considerable proportion reported postponing or avoiding dental visits, diagnostic procedures, and endodontic treatment. Furthermore, despite the relatively high educational level of the participants, similar concerns were still reported, suggesting that educational level alone may not necessarily reduce perceived risks related to dental care during pregnancy. From a behavioral perspective, this discrepancy may be interpreted cautiously in light of commonly used health behavior frameworks such as the Health Belief Model, which suggest that health-related actions may be influenced not only by knowledge but also by perceived risks and perceived barriers [28]. In the present study, participants frequently reported perceived risks and barriers regarding the safety of dental procedures, including local anesthesia, radiographic imaging, and medication use during pregnancy. Previous studies suggest that targeted educational interventions by dentists addressing perceived risks and correcting common misconceptions about dental radiography, anesthesia, and endodontic treatment during pregnancy are essential [12,29,30].

Multivariate analysis provided additional insights into the factors associated with participants’ perceptions and attitudes. In the present study, increasing age was associated with a higher likelihood of analgesic use for dental pain and with perceiving dental radiography during pregnancy as safe. Similar observations have been reported in a study by Barbieri et al., where older pregnant women tended to demonstrate greater awareness of oral health issues and were more likely to utilize dental services compared with younger women [31]. Education level was also related to perceptions regarding the safety of dental radiography and local anesthesia during pregnancy, which is consistent with earlier research indicating that higher educational attainment among pregnant women is often associated with better oral health knowledge and more favorable attitudes toward dental care during pregnancy [32,33]. In addition, the number of previous pregnancies was related to the reported reason for visiting a dentist during pregnancy. Previous studies have likewise suggested that pregnancy-related experience may influence oral health awareness and patterns of dental care utilization among pregnant women [31,32,33].

Shaping pregnant women’s attitudes toward oral health and promoting timely dental treatment could be enhanced through the active involvement of healthcare professionals. Dental service utilization by pregnant women may increase when oral health is emphasized during prenatal care through patient education, referrals, and active assessment by healthcare providers [2]. Nevertheless, oral health often remains underprioritized by antenatal care providers in routine prenatal consultations [34]. As a result, many pregnant women who experience oral health problems either delay seeking dental care or avoid dental visits altogether. In line with previous studies, the present findings suggest that routine dental attendance tends to decrease during pregnancy, even among women who had regularly attended dental check-ups before pregnancy [2]. This pattern of hesitation and concerns about the safety of dental procedures during pregnancy among pregnant women may be related to absence of clear information or adequate reassurance from antenatal care providers and dentists. However, the present study did not directly assess whether participants had received oral health information or counseling from healthcare providers.

Taken together, the findings of the present study have implications for clinical practice, public health strategies, and health policy aimed at improving oral healthcare utilization during pregnancy. In clinical practice, dentists and antenatal care providers may play an important role in addressing misconceptions, providing reassurance about dental care during pregnancy, and encouraging timely dental consultations through clear communication during prenatal visits [2]. At the population level, integrating oral health education and counseling into routine antenatal care and developing educational initiatives targeting both pregnant women and healthcare providers may help improve awareness and reduce treatment avoidance [2,12,29,30,35]. At the policy level, incorporating oral health assessments into standard prenatal care guidelines and strengthening referral pathways between prenatal care services and dental clinics may facilitate earlier identification of oral health needs and promote more appropriate use of dental services during pregnancy [2]. Together, these approaches may support improved access to dental care and contribute to better maternal oral health outcomes [7,35].

This study has several limitations. The use of convenience sampling and the relatively low response rate (130/500; 26%) may limit the generalizability of the findings and introduce potential non-response bias. Although the overall sample size was sufficient for descriptive analyses, some subgroup comparisons involved relatively small numbers, which may affect the stability of the statistical estimates. Due to the cross-sectional design of the study, causal relationships cannot be established, and the findings should be interpreted as associations between variables. The use of an online survey distributed through social media groups may have introduced selection bias, as women who actively participate in online communities may differ in health awareness compared with the general pregnant population. Also, the reliance on self-reported data may not fully reflect participants’ actual clinical oral health status. In addition, the study did not assess whether participants had received oral health information or counseling from healthcare providers during pregnancy, which may also influence dental care-seeking behavior. Future studies with larger and more diverse samples from different regions may allow more comprehensive multivariable analyses and more stable estimates.

## 5. Conclusions

The present study suggests that a considerable proportion of pregnant women hesitate to seek dental care and postpone endodontic treatment despite recognizing the importance of managing dental pain and infection. These findings may highlight the potential importance of targeted oral health education focusing on the safety, appropriate timing, and clinical necessity of dental treatment during pregnancy. Improving patient-centered communication and reinforcing evidence-based guidance by antenatal care providers and dentists may reduce concerns, uncertainty, and unnecessary treatment delays, ultimately contributing to better maternal and fetal health outcomes. Further studies conducted during dental visits may help explore pregnant women’s perspectives and attitudes toward oral healthcare in greater depth.

## Figures and Tables

**Table 1:** Survey questions and corresponding answer choices used in the study.

Questions	Answer Options
1. How old are you?	____ years
2. Are you currently employed?	I am employed./I am not employed.
3. What is your educational level?	Elementary/Middle School High School University Master’s/Doctorate
4. How many times have you been pregnant?	First time Second time Third time Four or more times
5. Which trimester of pregnancy are you currently in?	1st Trimester (0–3 months) 2nd Trimester (4–6 months) 3rd Trimester (7–9 months)
6. Do you visit the dentist for regular check-ups?	Yes/No
7. Did you experience severe toothache during pregnancy?	Yes/No
8. Did you visit a dentist during pregnancy?	Yes/No
9. If yes, what was your main reason for visiting the dentist?	Endodontic Periodontal Surgical Restorative Prosthodontic Routine check-up Orthodontic
10. Do you think it is necessary to treat toothache and dental infections during pregnancy?	Yes/No
11. Have you received root canal treatment while pregnant?	Yes/No
12. Do you currently have a tooth that requires root canal treatment? (If no, skip to Question 14.)	Yes/No
13. I plan to postpone root canal treatment until after delivery.	Yes, I will postpone treatment. No, I will receive treatment during pregnancy.
14. At which stage of pregnancy do you think root canal treatment is safe?	1st Trimester 2nd Trimester 3rd Trimester Safe throughout pregnancy Not safe at any stage
15. Did you take antibiotics for toothache during pregnancy?	Yes/No
16. Did you take analgesics (painkillers) for toothache during pregnancy?	Yes/No
17. Dental X-rays, when performed with a lead apron, are safe during pregnancy.	Agree/Disagree
18. It is safe to use local dental anesthesia to numb the tooth during dental treatment during pregnancy.	Agree/Disagree

**Table 2:** Educational level of the study participants (n = 130).

Educational Level	n	%
Elementary/Middle School	2	1.54%
High School	4	3.08%
University Degree	88	67.69%
Master’s/Doctorate	36	27.69%

Data are presented as number of participants (n) and percentage (%).

**Table 3:** Number of previous pregnancies reported by the participants (n = 130).

Number of Pregnancies	n	%
First pregnancy	101	77.69%
Second pregnancy	27	20.77%
Third pregnancy	1	0.77%
≥4 pregnancy	1	0.77%

Data are presented as number of participants (n) and percentage (%).

**Table 4:** Distribution of participants according to pregnancy trimester (n = 130).

Pregnancy Trimester of Participants	n	%
1st Trimester	6	4.62%
2nd Trimester	22	16.92%
3rd Trimester	102	78.46%

Data are presented as number of participants (n) and percentage (%).

**Table 5:** Self-reported dental attendance and toothache experience of the participants.

Variable	Yes, n (%)	No, n (%)
Regular dental check-ups before pregnancy	61 (46.92%)	69 (53.08%)
Dental visit during pregnancy	34 (26.15%)	96 (73.85%)
Severe toothache during pregnancy	22 (16.92%)	108 (83.08%)

Data are presented as number of participants (n) and percentage (%).

**Table 6:** Distribution of reported dental complaints among participants who visited the dentist during pregnancy (multiple responses allowed; total responses = 43).

Main Complaint	n	%
Tooth ache	7	16.28%
Endodontic	1	2.33%
Periodontal	13	30.23%
Surgical	6	13.95%
Restorative	6	13.95%
Prosthodontic	1	2.33%
Routine check-up	1	2.33%
Orthodontic	8	18.60%

Data are presented as number of participants (n) and percentage (%).

**Table 7:** Participants’ perceptions and experiences regarding endodontic treatment during pregnancy (n = 130).

Statement/Variable	Response Category	n (%)
Treating toothache and dental infections during pregnancy is necessary.	Yes	120 (92.31%)
	No	10 (7.69%)
Received root canal treatment during pregnancy.	Yes	5 (3.85%)
	No	125 (96.15%)
Perceived safe stage of pregnancy for root canal treatment.	1st Trimester (0–3 months)	2 (1.54%)
	2nd Trimester (4–6 months)	49 (37.69%)
	3rd Trimester (7–9 months)	6 (4.62%)
	Safe throughout pregnancy	26 (20%)
	Not safe at any stage	47 (36.15%)

Data are presented as number of participants (n) and percentage (%).

**Table 8:** Multivariable Logistic Regression Analyses of Predictors of Dental Visit, Perceived Safety of Dental Procedures, and Analgesic Use During Pregnancy.

Outcomes	Variables	Odds Ratio (95% CI)	*p*-Value
Presence of dental visit during pregnancy (all reasons)	Age	0.434 (0.174–1.624)	0.537
	Employment status (Reference: Employed)	0.335 (0.165–1.836)	0.791
	University graduate (Reference: non-university graduate)	0.416 (0.210–1.041)	0.191
	Number of pregnancies	1.190 (1.010–2.575)	**0.048**
	Gestational month	1.23 (0.54–2.79)	0.625
Perceiving root canal treatment as safe during pregnancy	Age	0.965 (0.866–1.077)	0.527
	Employment status (Reference: Employed)	0.783 (0.074–8.227)	0.838
	University graduate (Reference: non-university graduate)	1.645 (0.892–2.987)	0.094
	Number of pregnancies	1.181 (0.557–2.508)	0.664
	Gestational month	0.858 (0.415–1.773)	0.679
Analgesic use due to dental pain	Age	1.20 (1.03–1.39)	**0.018**
	Employment status (Reference: Employed)	0.47 (0.08–2.83)	0.408
	University graduate (Reference: non-university graduate)	1.10 (0.63–1.92)	0.740
	Number of pregnancies	0.68 (0.20–2.31)	0.536
	Gestational month	1.64 (0.90–2.98)	0.108
Perceiving dental radiography as safe during pregnancy	Age	1.14 (1.01–1.29)	**0.047**
	Employment status (Reference: Employed)	0.24 (0.05–1.23)	0.087
	University graduate (Reference: non-university graduate)	0.57 (0.37–0.89)	**0.013**
	Number of pregnancies	0.93 (0.32–2.65)	0.886
	Gestational month	0.61 (0.31–1.18)	0.143
Perceiving dental anesthesia as safe during pregnancy	Age	1.08 (0.97–1.20)	0.152
	Employment status (Reference: Employed)	1.46 (0.30–7.18)	0.641
	University graduate (Reference: non-university graduate)	0.63 (0.43–0.94)	**0.022**
	Number of pregnancies	1.23 (0.53–2.81)	0.633
	Gestational month	1.03 (0.64–1.68)	0.891

Multivariable logistic regression analysis of factors associated with dental visit during pregnancy, perceived safety of dental procedures (root canal treatment, radiography, and anesthesia), and analgesic use due to dental pain. Results are presented as odds ratios (OR) with 95% confidence intervals (CI). Statistically significant values (*p* < 0.05) are indicated in bold.

**Table 9:** Factors associated with avoidance or postponement of dental treatment among pregnant women requiring root canal treatment (n = 33).

Variable	Category	Avoided or Postponed Treatment n (%)	Did Not Avoid or Postpone Treatment n (%)	*p*-Value
Education level	Elementary/Middle School	0 (0%)	1 (100%)	0.543
	High School	1 (50%)	1 (50%)	
	University Degree	17 (68%)	8 (32%)	
	Master’s/Doctorate	3 (60%)	2 (40%)	
Employment status	Employed	20 (66.7%)	10 (33.3%)	0.252
	Unemployed	1 (33.3%)	2 (66.7%)	
Received root canal treatment during pregnancy	Yes	1 (20%)	4 (80%)	**0.028**
	No	20 (71.4%)	8 (28.6%)	
Analgesic use for toothache	Yes	7 (41.2%)	10 (58.8%)	**0.006**
	No	14 (87.5%)	2 (12.5%)	
Antibiotic use for toothache	Yes	0 (0%)	2 (100%)	0.054
	No	21 (67.7%)	10 (32.3%)	
Knowledge of safe timing for elective dental treatment	Yes	5 (55.6%)	4 (44.4%)	0.555
	No	16 (66.7%)	8 (33.3%)	
Number of pregnancies	1	18 (64.3%)	10 (35.7%)	0.854
	≥2	3 (60%)	2 (40%)	
Regular dental visits	Yes	8 (50%)	8 (50%)	0.114
	No	13 (76.5%)	4 (23.5%)	
Experienced severe toothache during pregnancy	Yes	11 (50%)	11 (50%)	**0.021**
	No	10 (90.9%)	1 (9.1%)	
Belief that dental infection should be treated	Yes	15 (57.7%)	11 (42.3%)	0.171
	No	6 (85.7%)	1 (14.3%)	

Data are presented as n (%). Associations between variables and avoidance/postponement of dental treatment were analyzed using the Chi-square test. Statistically significant values (*p* < 0.05) are indicated in bold.

**Table 10:** Management of toothache during pregnancy among participants who required root canal treatment (n = 33).

Statement/Variable	Response	n (%)
Use of antibiotics for toothache during pregnancy	Yes	2 (1.54%)
	No	31 (98.46%)
Use of analgesics (painkillers) for toothache during pregnancy	Yes	17 (51.52%)
	No	16 (48.48%)
Plans to postpone root canal treatment until after delivery due to safety concerns	Yes	24 (72.73%)
	No	9 (27.27%)

Data are presented as number of participants (n) and percentage (%).

**Table 11:** Participants’ perceptions regarding the safety of procedures commonly used in endodontic treatment during pregnancy (n = 130).

Statement/Variable	Response Category	n (%)
Dental radiography (X-rays), when performed with a lead apron, is safe during pregnancy.	Agree	30 (23.08%)
	Disagree	100 (76.92%)
The use of local dental anesthesia during dental treatment is safe in pregnancy.	Agree	65 (50%)
	Disagree	65 (50%)

Data are presented as number of participants (n) and percentage (%).

## Data Availability

The data presented in this study are available on reasonable request from the corresponding author. The data are not publicly available due to ethical and privacy restrictions involving human participants.
